# A Rare Fatal Case of Hemophagocytic Lymphohistiocytosis With Central Pontine Myelinolysis in the Setting of Epstein-Barr Virus Reactivation

**DOI:** 10.7759/cureus.83720

**Published:** 2025-05-08

**Authors:** Alexander C Goodman, Austin J Burns, Ian Tullberg

**Affiliations:** 1 Medicine, Rocky Vista University College of Osteopathic Medicine, Parker, USA; 2 Family Medicine, University of Colorado Health Memoral Hospital System, Colorado Springs, USA

**Keywords:** allogeneic hematopoietic stem cell transplantation, bone marrow biopsy, central pontine myelinolysis (cpm), epstein–barr virus, hemophagocytic lymphohistiocytosis (hlh)

## Abstract

Hemophagocytic lymphohistiocytosis (HLH) is a rare hyperinflammatory disorder associated with infections, malignancies, autoimmune conditions, and inflammatory states. HLH is characterized by unregulated cytokine release and immune activation, leading to widespread tissue damage. In this case, we describe a male in his 30s who presented to the emergency department with concerns about sepsis after being diagnosed with mastoiditis a week earlier. Further investigation revealed persistent fevers, pancytopenia, hepatosplenomegaly, hyperferritinemia, and hypertriglyceridemia, fulfilling the diagnostic criteria for HLH. Additional serologic testing revealed a significantly elevated Epstein-Barr virus (EBV) viral load on PCR, with positive immunoglobulin G (IgG) and negative IgM, consistent with EBV reactivation. HLH in the setting of EBV reactivation is rare and may be associated with a poor prognosis. Central pontine myelinolysis is a commonly fatal neurologic condition that is rarely attributed to HLH but should be considered when other causes are ruled out such as electrolyte derangements and rapid correction.

## Introduction

Hemophagocytic lymphohistiocytosis (HLH) is a rare condition characterized by the hyperactivation of natural killer (NK) cells, CD8+ cytotoxic lymphocytes, and macrophages [1]. As a result of uncontrolled cytokine release and immune activation, HLH leads to tissue injury and eventual multiorgan failure and death. HLH is divided into primary and secondary forms. Those with the primary disease typically present early in childhood, while those with the secondary disease are associated with an acute illness or malignancy. While rare, secondary HLH is associated with a mortality rate of 30-40% and is most commonly associated with malignancy (30.7% of cases), infection (24.3%), and autoimmune conditions (20.8%) [2]. The immune dysregulation seen in HLH is characterized by persistent fevers, hepatosplenomegaly, cytopenias, elevated aminotransferases, elevated ferritin levels, and coagulopathy.

The most commonly associated infection is primary Epstein-Barr virus (EBV) infection, with EBV reactivation being a rare trigger for HLH that has been reported in a few case studies [3-6]. EBV has been shown to infect CD8+ T cells as well as NK cells. Infection with the virus leads to monoclonal expansion of these cell lines, contributing to the widespread tissue damage seen in HLH [7-9]. Additionally, the immune cells infected by EBV release massive amounts of inflammatory cytokines, leading to a ‘cytokine storm' and further tissue damage [10]. Given the systemic nature of the disease, complications of HLH are frequently encountered. The most common complications include acute respiratory distress syndrome (ARDS), myocarditis, microangiopathies, and liver failure, with hemodynamic collapse being the leading cause of death in patients with this disease. Many other complications, such as central pontine myelinolysis (CPM), have been recognized; however, these have only been described in a few case studies [11,12].

Despite advances in understanding HLH, cases involving EBV reactivation remain poorly characterized, particularly when complicated by rare neurologic manifestations like CPM. This case report seeks to bridge this knowledge gap by providing insights into the diagnostic and therapeutic challenges faced in managing such complex presentations. Our aim is to describe a unique presentation of HLH triggered by EBV reactivation and complicated by CPM, with the goal of improving recognition and management strategies for similar cases.

## Case presentation

Initial presentation

**Figure 1:**
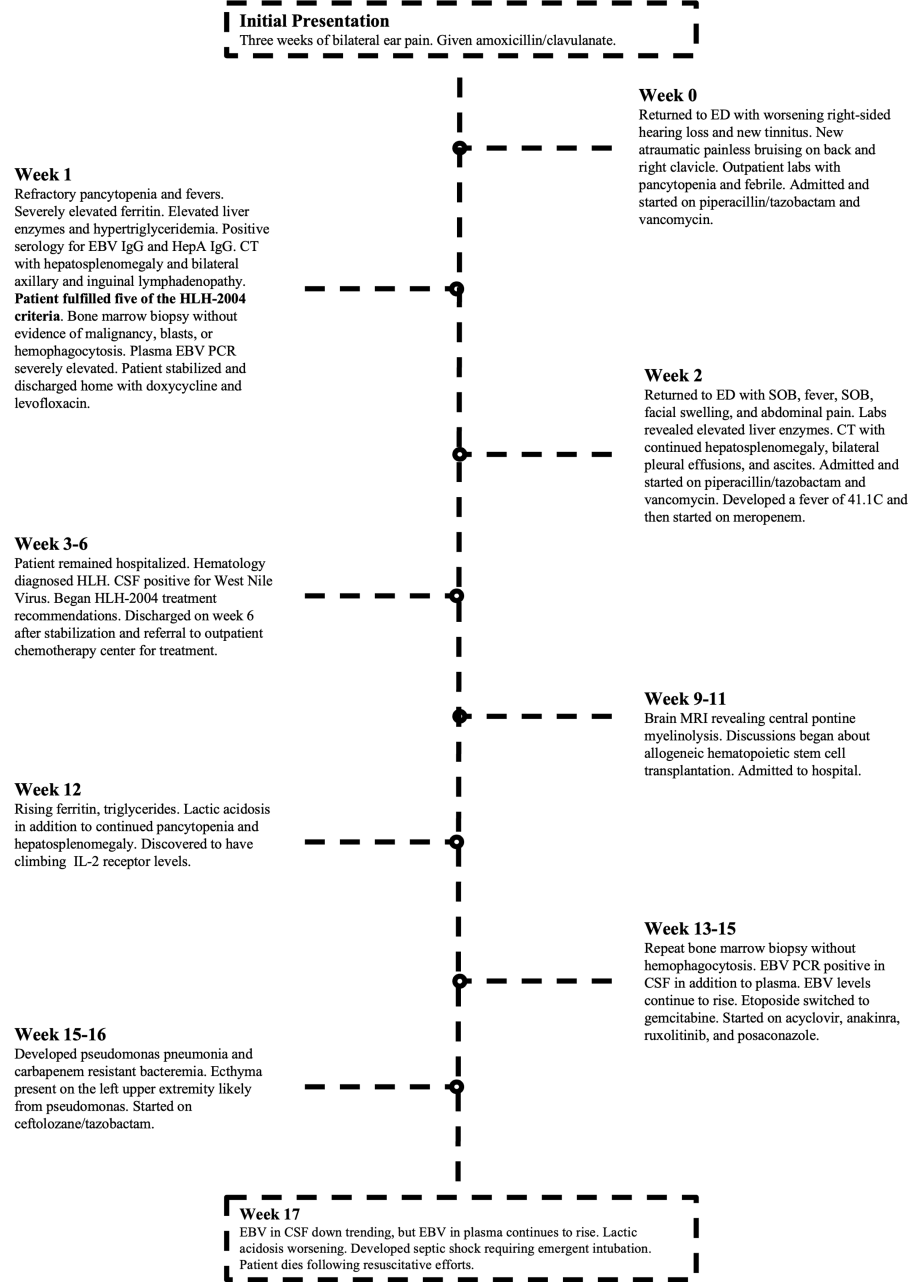
Timeline of our patient’s clinical course hemophagocytic lymphohistiocytosis (HLH); Epstein-Barr virus (EBV); immunoglobulin (Ig); hepatitis A (HepA); shortness of breath (SOB)

An active male in his 30s with no significant past medical history or prior hospitalizations initially presented to the emergency department with three weeks of progressively worsening bilateral ear pain. He was sent home with amoxicillin/clavulanate for suspected otitis media (Figure 1). Four days later, the patient returned to the same emergency department for worsening right-sided hearing loss, persistent fever, and new bilateral tinnitus. He also noted new, atraumatic, painless bruising on his back that his daughter had noticed a few days earlier. At that time, his vitals were notable for a fever of 38.6 °C (101.4 °F). Physical exam revealed a fatigued-appearing male with bilateral erythema and bulging of the tympanic membranes, and scattered, nontender, nickel-sized ecchymoses on his back and right clavicle. Lab studies demonstrated pancytopenia as well as hyponatremia (131 mEq/L), hypocalcemia (7.9 mEq/L), and hypoalbuminemia (3.2 g/dL). A CT scan of the head revealed bilateral mastoid effusions with a small amount of possible debris or fluid in the right middle ear cavity, consistent with mastoiditis; however, the patient had no tenderness to palpation near the mastoid process. He was admitted and started on piperacillin/tazobactam and vancomycin due to concerns for sepsis.

Investigations

**Figure 2:**
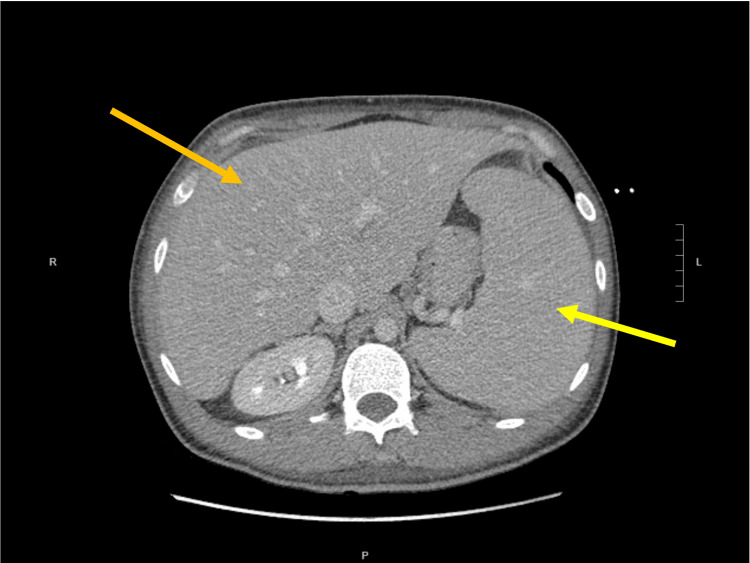
A CT scan of the abdomen and pelvis demonstrating hepatomegaly (orange arrow) and splenomegaly (yellow arrow) The spleen measured 16.2 cm in anterior-posterior diameter.

Following admission, more extensive blood work was performed, revealing several abnormalities, including hyperbilirubinemia, elevated aminotransferases, and hypertriglyceridemia. Infectious disease was consulted to investigate the pancytopenia and systemic symptoms that continued despite antibiotic therapy. The patient then underwent a CT of the abdomen and pelvis. He was found to have hepatosplenomegaly, prominent lymph nodes noted in the bilateral axillary and inguinal chains with normal morphology, and interlobular septal thickening in the lung apices (Figure 2). Hematology was consulted for concerns of a hematologic malignancy. Hematology recommended a bone marrow biopsy and peripheral flow cytometry. The bone marrow biopsy showed a normocellular pattern throughout with no increase in blasts, and the flow cytometry demonstrated no significant abnormalities.

The patient continued to experience fever and pancytopenia over the next couple of days. Aminotransferases began to trend upward while bilirubin remained stable. Iron studies showed a severely elevated ferritin (2,890 ng/mL), low serum iron (37 mcg/dL), and reduced total iron binding capacity (201 mcg/dL). His lactate dehydrogenase (LDH) level was elevated at 1,290 U/L. Throughout the hospitalization, the patient developed generalized joint aches. Given his purpuric rash and myalgias, there was a concern for an autoimmune process. However, the lab results returned with a negative autoimmune panel, including antineutrophil cytoplasmic antibodies (ANCA), Sjögren's syndrome (SS)-A, SS-B, ribonucleoprotein (RNP), anti-histidyl tRNA synthetase (anti-Jo-1), antinuclear antibody (ANA), and cyclic citrullinated peptide (CCP) (Table 1). Interleukin (IL)-2 receptor levels were found to be 1859 U/mL and 2952 U/mL (reference range 223-710 U/mL) shortly thereafter.

**Table 1:** Qualitative serologic and autoimmune testing results Serologic results listed as positive or negative reflect qualitative testing only. Quantitative values and reference ranges were not accessible from the source records. antinuclear antibody (ANA), anti-neutrophil cytoplasmic antibodies (ANCA), anti-Ro (anti-SS-A), anti-La (anti-SS-B), cerebrospinal fluid (CSF), cytomegalovirus (CMV), Epstein-Barr virus (EBV), human immunodeficiency virus (HIV), herpes simplex virus (HSV), rheumatoid factor (RF), varicella zoster virus (VZV)

Laboratory Test	Result	Positive Threshold
ANA	(-)	> 1:160 titer
ANCA	(-)	> 20 U/mL
anti-Jo-1	(-)	> 20 U/mL
anti-SS-A	(-)	> 20 U/mL
anti-SS-B	(-)	> 20 U/mL
Bartonella IgG	(-)	> 1:256 titer
Blastomycosis IgG	(-)	> 1:8-1:32 titer
Brucella IgG	(-)	> 10-12 U/mL
Brucella IgM	(-)	> 10-12 U/mL
CMV IgG	(-)	> 1.10 arbitrary units
CMV IgM	(-)	> 1.10 arbitrary units
Coccidioides IgG	(-)	> 1:2 titer
COVID-19 PCR	(-)	N/a
Coxsackie A IgG	(+)	> 1.10 arbitrary units
Coxsackie A IgM	(-)	> 1.10 arbitrary units
Coxsackie B IgG	(+)	> 1.10 arbitrary units
Coxsackie B IgM	(-)	> 1.10 arbitrary units
EBV IgG	(+)	> 1.10 arbitrary units
EBV IgM	(-)	> 1.10 arbitrary units
EBV viral load	137,000^+^ IU/mL	> 700 IU/mL
Ehrlichiosis IgG	(-)	> 1:64 titer
Enterovirus PCR	(-)	N/a
Histoplasmosis IgG	(-)	> 1:32 titer
HIV PCR	(-)	> 20-50 copies/mL
HSV PCR (CSF)	(-)	N/A
Lyme IgG	(-)	> 1.10 arbitrary units
Mycoplasma IgG	(-)	> 1:64-1:128 titer
Parvovirus B19 IgG	(-)	> 1.10 arbitrary units
RF	(-)	> 14-20 IU/mL
Rickettsiosis IgG	(-)	> 1:128
VZV IgG	(-)	> 135 AU/mL
VZV IgM	(-)	> 110 AU/mL
West Nile Virus PCR	(-)	N/a

At this point in the patient’s clinical course, he and his wife became overwhelmed with anxiety due to the uncertainty of his condition. Upon further questioning, the patient revealed he has several goats, pigs, and chickens at home and admitted to frequently drinking filtered, non-pasteurized goat milk. Given the high-risk exposures and continued refractory fever, the patient underwent extensive serology testing (Table 1). The infectious serology testing largely returned unremarkable; however, EBV PCR had significantly elevated levels (137,000 IU/mL). Given the positive EBV PCR and IgG but negative IgM, it was determined that the patient was experiencing an EBV reactivation. Lastly, a fungal panel was performed, revealing no active fungal infections at the time (Table 1).

Differential diagnosis

The original differential diagnosis during his first hospitalization included mastoiditis, sepsis, Ramsay Hunt syndrome, viral aplastic anemia, thrombotic thrombocytopenic purpura, leukemia, lymphoma, hemophagocytic lymphohistiocytosis, systemic mastocytosis, and adult-onset systemic juvenile idiopathic arthritis. The patient presented with bilateral ear pain, a fever, and pancytopenia. A head CT led to a diagnosis of mastoiditis; however, the hematologic abnormalities remained unaccounted for.

Subsequent investigations led to the discovery of neck, axillary, and inguinal lymphadenopathy, along with an erythematous, non-ecchymotic rash across the upper back and chest. At this time, the fever and headaches failed to improve despite intravenous piperacillin/tazobactam and vancomycin. Lab work revealed elevated LDH, hyperferritinemia, normal haptoglobin, low reticulocyte count, and negative infectious titers except for EBV. This narrowed the differential list to lymphoma, leukemia, systemic mastocytosis, and HLH.

The initial bone marrow biopsy on the patient’s first hospitalization showed no evidence of hemophagocytosis or abnormal blasts. Combined with a normal peripheral smear, HLH, lymphoma, leukemia, and systemic mastocytosis were considered less likely. However, the presence of hemophagocytosis on bone marrow biopsy is neither sensitive nor specific for diagnosing HLH [13,14]. 

At the time of our patient’s first admission, he had a fever, CT-proven splenomegaly (Figure 2), pancytopenia, hypertriglyceridemia, and hyperferritinemia; therefore, he had fulfilled five of the HLH-2004 diagnostic criteria early on in his initial hospital course (Table 2) [14,15]. Despite this, a diagnosis was not rendered by the covering hematology team. After readmission, the covering hematology team promptly recognized that the patient met HLH-2004 criteria and made the diagnosis, three weeks after the patient’s initial presentation. Further laboratory work showed increased IL-2 receptor levels, further supporting the diagnosis of HLH. 

**Table 2:** HLH-2004 criteria in comparison to our patient’s status The guidelines require the presence of at least five of the eight criteria to make the clinical diagnosis of HLH. Criteria A or Criteria B must be fulfilled for a diagnosis to be made. The following reference ranges were used: WBC: 4.0-11.0 ×10³/µL, HgB: 13.5-17.5 g/dL, PLT: 150-400 ×10³/µL, triglycerides: < 150 mg/dL, fibrinogen: 200-400 mg/dL, fibrinogen: 24-336 ng/mL, and CD25: 223-710 U/mL. hemophagocytic lymphohistiocytosis (HLH)

HLH-2004 Criteria		Our Patient on Presentation
A. A molecular diagnosis consistent with HLH *OR*		
B. Any 5 of the 8 following criteria:		
	Fever	Present
	Cytopenia (≥ 2 cell lines)	Present (WBC: 1.8 x10^3^/uL, HgB: 11.5 g/dL, PLT: 107 x 10^3^/uL)
	Splenomegaly	Present
	Hypertriglyceridemia/hypofibrinogenemia	Present (Triglycerides: 221 mg/dL)
	Hemophagocytosis in any tissue	Not present
	Low or absent natural killer cell activity	Not present
	Serum ferritin ≥ 500 μg/L	Present (Ferritin: 2,890 ng/mL)
	Soluble CD25 (IL-2 receptor) ≥ 2400 U/mL	Present (CD25: 2,952 U/mL

TREATMENT

Without treatment, patients with HLH can rapidly deteriorate. Treatment is focused on immunosuppression and cytotoxic-targeted chemotherapy with the goal of preventing any life-threatening inflammatory processes. The HLH-94 guidelines outlined the first protocol to treat HLH, including dexamethasone, etoposide, cyclosporine A, and intrathecal methotrexate [16]. The HLH-2004 guidelines revisited this protocol. The most up-to-date guidelines left the protocol largely intact; however, they now include the addition of intrathecal steroids alongside methotrexate [14,17]. After the diagnosis of EBV-HLH was established, the patient was immediately started on IV etoposide (150 mg/m^2^), IV dexamethasone (10 mg/m^2^), IV cyclosporin A (200 μg/L), and intrathecal methotrexate.

After several weeks on the treatment protocol, the patient developed a severe headache, followed by aphasia and encephalopathy characterized by confusion and hallucinations. An MRI revealed increased signal intensity within the central pons on T2-weighted images, along with a small patchy focus of increased signal in the left putamen. Based on these findings and the presence of hyponatremia, with sodium levels as low as 126 mEq/L throughout his treatment, it was determined that the patient had developed central pontine myelinolysis (CPM). Given that his hyponatremia was managed conservatively, the development of CPM was believed to be secondary to HLH itself rather than rapid sodium correction. Following the diagnosis of CPM, the patient was initiated on intravenous rituximab at 375 mg/m² weekly for four weeks.

Despite being on the HLH-2004 treatment protocol for several weeks, the patient continued to decline. A repeat bone marrow biopsy revealed no evidence of hemophagocytosis. Newer therapies are focused on immunomodulation and include anakinra (IL-1 blocker), ruxolitinib (JAK1/2 inhibitor), alemtuzumab (CD52 monoclonal antibody), and emapalumab (anti-IFN-y monoclonal antibody) [10,18-20]. These are not considered standard of care but show potential as a treatment option and can be considered in refractory cases. Due to the lack of treatment response, he was started on Anakinra 100 mg daily and ruxolitinib 15 mg twice daily. Shortly thereafter, it was discovered that the reactivated EBV had disseminated into his cerebrospinal fluid (CSF). Intrathecal rituximab was added to his treatment regimen for a duration of four weeks. Despite these therapies, EBV titers and HLH markers continued to rise. The IV etoposide was discontinued to stay under the maximum cumulative dose of 2 to 3 g/m^2^ [17]. IV gemcitabine 1 g/m^2^ was started after the etoposide was discontinued. Allogeneic hematopoietic stem cell transplantation was considered but could not be pursued due to the patient’s deteriorating clinical status.

Outcome and follow-up

In the weeks following the initiation of the HLH treatment protocol, the patient was in and out of the hospital with several disease- and treatment-related complications (Figure 1). These complications included West Nile virus encephalitis, *Pseudomonas* pneumonia and bacteremia, left upper extremity cellulitis, atrial fibrillation, ecthyma, severe lactic acidosis, enteritis, splenic infarct, and liver failure.

Despite intensive chemotherapeutic treatment and immunosuppressants, the patient’s EBV titers continued to rise. Other serum markers of HLH remained elevated as well, including ferritin, triglycerides, and IL-2 receptor levels. Notably, his LDH levels remained elevated. The patient began developing worsening black, necrotic lesions on his left upper extremity. Ultimately, he developed pneumonia with *Pseudomonas aeruginosa,* and carbapenem-resistant bacteremia was discovered on blood culture. He was started on IV ceftolozane/tazobactam. Despite continued antibiotic therapy, he began experiencing respiratory distress requiring emergent intubation. He experienced cardiac arrest shortly after and was unresponsive despite 40 minutes of resuscitative efforts, including CPR, defibrillation, and cardiac resuscitative medications.

A final bone marrow biopsy was performed at the time of autopsy, confirming the presence of hemophagocytosis. Additionally, the autopsy confirmed EBV-driven hepatitis with atypical lymphocytes. The spleen also showed atypical lymphocytes with visible hemophagocytosis. In addition to the hematologic findings, the patient was discovered to have multifocal pneumonia, worse in the left upper lobe, along with infarcts of the liver with centrilobular necrosis, splenic infarct, global cerebral hypoxia, and central white matter demyelination, consistent with CPM. The cause of death was officially determined to be septic shock with severe metabolic acidosis from lactic acidosis, likely related to infectious etiology in an immunosuppressed patient undergoing treatment for EBV-driven HLH.

## Discussion

The incidence rate of HLH has been increasing in recent years, with the majority of diagnoses made in urban hospitals and academic centers [2,21]. This trend highlights the need for increased awareness and diagnostic capacity in rural areas, where such conditions may go unrecognized. This suggests that HLH is underdiagnosed in rural areas, as urban hospitals are better equipped to test for rare diseases. Nonetheless, an HLH diagnosis can often be made with a thorough physical exam and routine blood work, although imaging can be helpful. Bone marrow biopsies may be helpful by demonstrating hemophagocytosis or other related processes, but they are neither sensitive nor specific in diagnosing HLH. Prompt recognition and treatment are critical to reducing HLH-associated morbidity and mortality. According to HLH-2004 diagnostic criteria, a biopsy with evidence of hemophagocytosis is not required for the diagnosis of HLH; rather, it is just one of eight diagnostic criteria (Table 2) [15]. In this case, the diagnosis of HLH could have been identified solely based on the physical exam and blood work, allowing for earlier initiation of treatment and potentially improving the outcome. This case underscores the importance of recognizing EBV reactivation as a possible HLH trigger in patients with unexplained fevers and cytopenias. Additionally, rare complications may occur even in the absence of traditional risk factors, as demonstrated by this patient’s development of CPM despite appropriate correction of his hyponatremia. By improving early identification and treatment of HLH, clinicians may help reduce the poor outcomes often associated with the disease.

**Figure 3:**
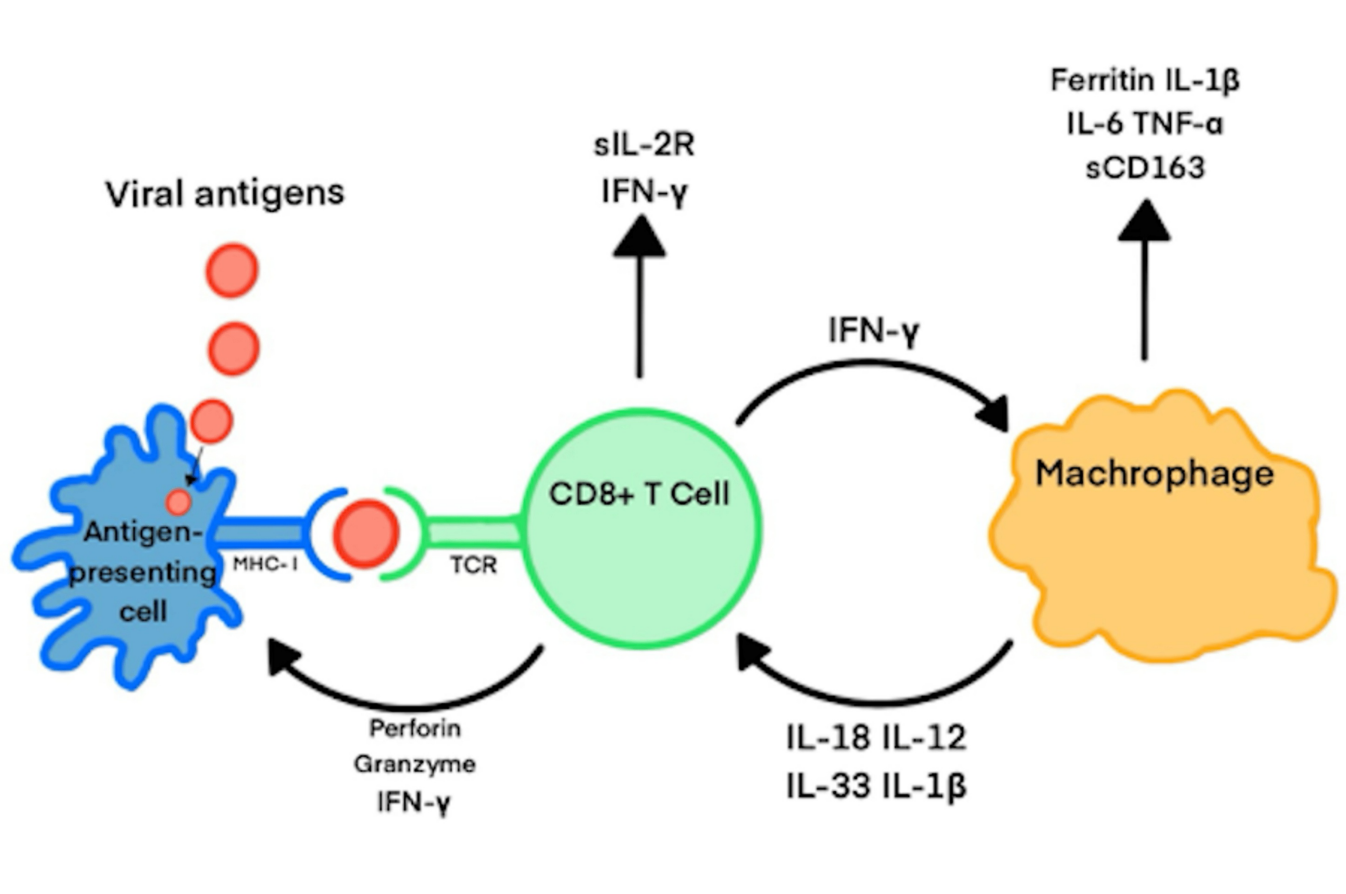
The proposed cellular and molecular mechanism behind HLH. Briefly, the process begins with an antigen, such as Epstein–Barr virus (EBV) in this case, being presented to a CD8+ T cell. This triggers T cell activation and the release of interferon-gamma (IFN-γ), which subsequently activates macrophages. The activated macrophages further stimulate CD8+ T cells, establishing a positive feedback loop that drives an uncontrolled immune response and leads to widespread tissue damage. The figure is the author's own creation. Figure Credits: Austin Burns, created using Microsoft PowerPoint

In EBV-HLH, the pathophysiology is likely multifactorial. It is thought that toll-like receptor activation by EBV antigens leads to the overproduction of inflammatory mediators and cytokines, resulting in uncontrolled CD8+ T-cell activation (Figure 3). However, the exact mechanism remains unclear, as most EBV-HLH interactions have been studied only in case reports. It is thought that in EBV-HLH specifically, the inability to clear the viral infection leads to sustained CD8+ T cell activation, which is amplified by IFN-γ and MHC-I upregulation. The continued release of IFN-γ from CD8+ T cells activates macrophages to release several inflammatory mediators and cytokines, including IL-6, IL-1β, sCD163, TNFα, IL-18, IL-12, and IL-33, further enhancing IFN-γ release and perpetuating the immune response [8,22,23]. In vitro studies using both cell lines and animal models are needed to further characterize the exact mechanism behind the monoclonal expansion and excessive cytokine release seen in EBV-triggered HLH.

A review of the literature identified three case reports describing patients who developed CPM in the setting of HLH and without other known risk factors [11,12,24]. The precise pathophysiology of CPM due to HLH remains poorly understood. One theory suggests that hypercytokinemia from the EBV-infected immune cells disrupts the osmotic regulation in the brain, leading to demyelination and the neurologic sequelae characteristic of CPM [12,25,26]. In this case, the absence of severe electrolyte disturbances or traditional risk factors, such as alcoholism, burns, or liver transplantation, supports HLH as a plausible cause. While this growing body of evidence suggests a potential link, some studies argue the association may be coincidental [12]. Further studies are needed to investigate the link between HLH and CPM, specifically looking at osmotic regulation, the relationship between the blood-brain barrier, and hypercytokinemia.

Treatment of HLH is uniquely difficult due to the high variability in its underlying causes. The HLH Steering Committee of the Histiocyte Society has recommended using the HLH-94 protocol in treating both primary and secondary HLH [27]. Although this protocol was originally developed for familial HLH, it is commonly applied in secondary cases. The HLH-94 protocol includes dexamethasone, IV etoposide, PO cyclosporine A (CSA), and intrathecal methotrexate. This protocol showed a three-year probability of survival of 55% [16]. While this has improved the overall survival rate, more tailored treatment regimens are likely more effective depending on the HLH subtype.

Treating EBV-HLH remains particularly challenging and is associated with poorer outcomes. One study reported an overall worse 5-year survivability (25.1%) when compared to autoimmune (82.4%), other infections (78.7%), and unknown causes (55.5%) of HLH [28]. Some studies have proposed the use of B-cell-specific targets, such as rituximab or alemtuzumab, for the treatment of EBV-HLH [29,30], but insufficient data currently prevent broad recommendations. Once patients with HLH begin to show signs of remission, they can be considered for allogeneic hematopoietic stem cell transplantation (HSCT), which remains the only curative option [31]. Typically, HSCT is reserved for patients once remission has begun, as those with active HLH disease who undergo HSCT have been shown to have worse outcomes [32]. However, in EBV-HLH, HSCT should be considered in those with increasing or sustained levels of high EBV DNA titers [33,34].

This case presents a rare and detailed clinical trajectory of EBV-triggered HLH complicated by CPM in a previously healthy young adult. It contributes to the limited literature connecting EBV reactivation, HLH, and CPM in adult patients. Furthermore, it emphasizes the importance of early recognition and aggressive management. However, the report is limited by the lack of definitive causal evidence linking HLH to CPM, and by potential delays in diagnosis. Additionally, the absence of quantitative serologic data for many infectious and autoimmune tests limits the ability to fully characterize the case.

## Conclusions

This case report details a rare and fatal presentation of hemophagocytic lymphohistiocytosis (HLH) triggered by Epstein-Barr virus (EBV) reactivation and complicated by central pontine myelinolysis (CPM). A male in his 30s initially presented with ear pain and was diagnosed with mastoiditis, but subsequent evaluation revealed pancytopenia, hepatosplenomegaly, hyperferritinemia, and elevated IL-2 receptor levels, meeting the HLH-2004 diagnostic criteria. Despite initiation of the appropriate treatment protocol and later immunomodulatory therapies, the patient’s condition deteriorated, with persistent EBV viremia and neurologic decline due to CPM. He ultimately succumbed to septic shock and lactic acidosis despite aggressive treatment.

While the HLH-2004 criteria were met early in the hospital course, the atypical presentation with mastoiditis highlights the diagnostic complexity clinicians may encounter. This case underscores the diagnostic challenges of HLH as well as the need for increased awareness of HLH, particularly in settings where diagnostic delays may exacerbate poor outcomes. The poor prognosis associated with EBV-HLH, compounded by the rare but serious neurologic complication of CPM, reinforces the importance of maintaining a high index of suspicion for HLH in a patient with unexplained fevers and cytopenias. Future in vitro studies and longitudinal analyses of cytokine profiles in HLH patients who develop neurologic complications may help clarify the mechanisms underlying CPM. These insights could inform novel therapeutic strategies and potentially improve outcomes in refractory cases such as the one presented here.
